# A novel method to improve the osteogenesis capacity of hUCMSCs with dual‐directional pre‐induction under screened co‐culture conditions

**DOI:** 10.1111/cpr.12740

**Published:** 2019-12-09

**Authors:** Qiong Rong, Shuyi Li, Yang Zhou, Yuanming Geng, Shangbin Liu, Wanqiu Wu, Tim Forouzanfar, Gang Wu, Zhiyong Zhang, Miao Zhou

**Affiliations:** ^1^ Key Laboratory of Oral Medicine Guangzhou Institute of Oral Disease Affiliated Stomatology Hospital of Guangzhou Medical University Guangzhou China; ^2^ Department of Stomatology The First People's Hospital of Yunnan Province The Affiliated Hospital of Kunming University of Science and Technology Kunming China; ^3^ Department of Oral and Maxillofacial Surgery/Pathology Amsterdam UMC and Academic Center for Dentistry Amsterdam (ACTA) Vrije Universiteit Amsterdam Amsterdam Movement Science Amsterdam The Netherlands; ^4^ Department of Stomatology Zhujiang Hospital Southern Medical University Guangzhou China; ^5^ Department of Oral Implantology and Prosthetic Dentistry Academic Center for Dentistry Amsterdam (ACTA) University of Amsterdam and Vrije Universiteit Amsterdam Amsterdam The Netherlands; ^6^ Translational Research Centre of Regenerative Medicine and 3D Printing Technologies of Guangzhou Medical University The Third Affiliated Hospital of Guangzhou Medical University Guangzhou China

**Keywords:** bone defect, co‐culture, dual‐directional differentiation, human umbilical cord mesenchymal stem cells, osteogenesis

## Abstract

**Objectives:**

Mesenchymal stem cells (MSCs) based therapy for bone regeneration has been regarded as a promising method in the clinic. However, hBMSCs with invasive harvesting process and undesirable proliferation rate hinder the extensive usage. HUCMSCs of easier access and excellent performances provide an alternative for the fabrication of tissue‐engineered bone construct. Evidence suggested the osteogenesis ability of hUCMSCs was weaker than that of hBMSCs. To address this issue, a co‐culture strategy of osteogenically and angiogenically induced hUCMSCs has been proposed since thorough vascularization facilitates the blood‐borne nutrition and oxygen to transport in the scaffold, synergistically expediting the process of ossification.

**Materials and methods:**

Herein, we used osteogenic‐ and angiogenic‐differentiated hUCMSCs for co‐culture in screened culture medium to elevate the osteogenic capacity with in vitro studies and finally coupled with 3D TCP scaffold to repair rat's critical‐sized calvarial bone defect. By dual‐directional induction, hUCMSCs could differentiate into osteoblasts and endothelial cells, respectively. To optimize the co‐culture condition, gradient ratios of dual‐directional differentiated hUCMSCs co‐cultured under different medium were studied to determine the appropriate condition.

**Results:**

It revealed that the osteogenic‐ and angiogenic‐induced hUCMSCs mixed with the ratio of 3:1 co‐cultured in the mixed medium of osteogenic induction medium to endothelial cell induction medium of 3:1 possessed more mineralization nodules. Similarly, ALP and osteogenesis/angiogenesis‐related genes expressions were relatively higher. Further evidence of bone defect repair with 3D printed TCP of 3:1 group exhibited better restoration outcomes.

**Conclusions:**

Our work demonstrated a favourable and convenient approach of dual‐directional differentiated hUCMSCs co‐culture to improve the osteogenesis, establishing a novel way to fabricate tissue‐engineered bone graft with 3D TCP for large bone defect augmentation.

## 1 INTRODUCTION

Large bone defects resulting from trauma, infection, tumour resection or congenital deformities severely affect the original contours and functions. Autologous bone grafts, routinely regarded as the “golden standard” for bone transplantation, require a second surgical site, inevitably causing new tissue damages and bringing seriously physical, psychological and economic burdens to patients.^1^ To attain non‐invasive and much safer customized rehabilitation of bone defects, mesenchymal stem cells (MSCs) have been suggested to facilitate the fabrication of customized, bioactive tissue‐engineered bone grafts based on the bone scaffold made by three‐dimensional printing (3DP), so as to provide promising evidence for clinical settings.^2^, ^3^ To date, with the in‐depth study, MSCs are extensively used as seed cells for bone tissue regeneration, especially MSCs isolated from bone marrow, characterize better osteogenic differentiation capacity and are frequently used in bone tissue engineering.^4^ However, the acquisition number of human bone marrow mesenchymal stem cells (hBMSCs) cannot meet the huge demands for bone repair with invasive process, and the cellular activities are much influenced by the donor's age and health status, greatly restricting the clinical application.^5^ In contrast, MSCs from the Wharton's jelly of the neonatal umbilical cord, named human umbilical cord mesenchymal stem cells (hUCMSCs), possess a higher cell yield, rapid proliferation capacity, amounts of secreted cytokines related to cell migration, inflammation, immune regulation, angiogenesis (vascular endothelial growth factor [VEGF], insulin‐like growth factor‐1, transforming growth factor and platelet‐derived growth factor [PDGF]), neurogenic and wound healing processes.^6^, ^7^, ^8^ Above all, hUCMSCs do not express the major histocompatibility complex II (MHC II) and immunoregulatory factor B7 costimulatory molecules, which are responsible for the alloimmune response.^9^ Therefore, it is believed that hUCMSCs own the characteristics of both seed cells and cytokines in the process of fabrication of tissue‐engineered bone, which will be a promising choice to enhance the bioactivity of tissue‐engineered bone graft.^10^ Numerous researches have also shown that hUCMSCs have superior osteogenic properties attached to the scaffolds and are suitable seed cells for bone tissue engineering.^11^

Given that some studies hold the osteogenic ability of hUCMSCs is weaker than that of hBMSCs, it is worthy to improve the osteogenesis ability of hUCMSCs.^12^ Besides, due to the lack of nutrition and oxygen supply, the closer MSCs to the centre of the scaffolds, the harder to survive. Studies demonstrated that within 200 μm distance from the blood vessels is critical for the survival and retention of viable cells.^13^, ^14^ Considering that VEGF plays a vital role in facilitating angiogenesis as well as stimulating osteogenesis by regulating related growth factors, osteoblasts, BMSCs, hUCMSCs or human adipose mesenchymal stem cells (hADMSCs) derived VEGF is much emphasized.^8^, ^15^, ^16^, ^17^, ^18^ An optimal concentration of VEGF is required for angiogenesis and osteogenesis coupling for intramembranous ossification on account that a higher or lower concentration of VEGF could result in compromised bone formation.^17^ In light of these phenomena, a co‐culture system of osteogenic cells (stem cells, osteoblasts, etc) and angiogenic cells (endothelial progenitor cells, endothelial cells, etc) has been raised, which has been validated there is a synergistic effect in promoting osteogenesis and angiogenesis, favourable for the survival and prognosis of osteoblasts in the scaffolds, thus ensuring a successful ossification of tissue‐engineered bone.^19^, ^20^ Nevertheless, the culture of endothelial progenitor cells and endothelial cells in vitro is difficult, and the number of isolated cells is very less with limited proliferation ability.^21^ Besides, in the clinical application of co‐culturing these two kinds of cells, the risks of immune rejection and disease transmission will increase for the different cell sources. As a result, to lower the aforementioned risks, one single kind of MSCs with abundant sources should be applied to differentiate into osteoblasts and endothelial cells, respectively, before sent for co‐culture. To date, studies concerning dual‐directional differentiation of one single kind of stem cell are scarce.

Studies show that the mixing ratio of the two kinds of cells and the culture medium used for co‐culture directly influence the outcome of osteogenesis.^22^ On account of different cell types and treatment methods used by the researchers, no acceptable standard of co‐culture has been recognized so far. Most researchers prefer to adopt the method of mixing two types of cells with the ratio of 1:1, cultured in the osteogenic induction medium or the co‐culture medium (50% osteogenic induction medium and 50% endothelial growth medium).^20^, ^23^, ^24^ By far, there has been no report elaborating on the culture condition concerning the bi‐directional differentiation of hUCMSCs.

In this study, hUCMSCs were pre‐induced to differentiate into osteoblasts and endothelial cells, respectively, and then co‐cultured under different ratios in the screened culture medium. By comparing the rehabilitation effects of critical‐sized calvarial bone defect with tissue‐engineered bone graft fabricated by incorporating different ratios of induced cells onto 3D printed tricalcium phosphate (TCP) scaffold, the osteogenesis outcome of the co‐cultured cells from bi‐directional differentiation of pre‐induced hUCMSCs was explored and analysed, so as to provide a state‐of‐the‐art method for the application of hUCMSCs in bone tissue engineering.

## 2 MATERIALS AND METHODS

### 2.1 Isolation, cultivation and identification of hUCMSCs

The experiment design and examinations of this study were briefly illustrated (Figure S1). With the informed consents from three volunteers, the umbilical cords were obtained for cell isolation and histological observation. Briefly, after sterilization and thorough washing, the umbilical vein, two arteries and umbilical cord envelope were removed, and the Wharton's jelly was obtained. After cut into pieces, 0.1% type II collagenase (Sigma‐Aldrich) was used for digestion overnight at 4°C. Then, the tissue suspension was screened, plated in mesenchymal stem cells medium (SM, Table 1) and incubated in 5% CO_2_ at 37°C. After 3 days, the adherent growth of hUCMSCs was observed. Passage 4‐8 was chosen for subsequent experiments.

**Table 1:** Culture medium

Abbreviated name	Full name	Components	Brand/Country
SM	Mesenchymal stem cells medium	MGro‐500 chemically defined MSC medium (MesenGro basal medium, 450 mL; MesenGro supplement 50 mL)	StemRD/USA
PM	Proliferation medium	High glucose Dulbecco's modified eagle medium 10% foetal bovine serum (FBS) 1% penicillin‐streptomycin	Gibco/USA
OM	Osteogenic medium	PM 100 nmol/L dexamethasone 10 mmol/L β‐glycerophosphate 50 μg/mL L‐ascorbic acid	Solarbio/China Sigma/Germany Sigma/China
EGM	Endothelial cell growth medium‐2	Endothelial cell basal medium‐2 EGM‐2 BulletKit (10 mL FBS, 0.2 mL hydrocortisone, 2.0 mL hFGF‐B, 0.5 mL VEGF, 0.5 mL ascorbic acid, 0.5 mL hEGF, 0.5 mL GA‐1000, 0.5 mL Heparin)	Lonza/USA Lonza/USA
EIM	Endothelial cell induction medium	EGM 25 ng/mL human VEGF 10 ng/mL human bFGF	PeproTech/USA PeproTech/USA
EMM	Equivalent mixed medium (OM/EIM)	1:1 mixture of OM and EIM	/
PMM	Proportional mixed medium (OM/EIM)	OM:EIM = os‐hUCMSCs:en‐hUCMSCs	/

For identification, 6 × 10^5^ hUCMSCs in PBS were incubated with FITC‐labelled mouse anti‐human IgG1 kappa isotype (eBioscience), mouse anti‐human CD73 (eBioscience) and mouse anti‐human CD90 (eBioscience), PE‐labelled mouse anti‐human IgG1 kappa isotype (Santa Cruz), mouse anti‐human CD34 (Santa Cruz) and CD45 (Santa Cruz), respectively, on ice for 30 minutes. Flow cytometry (BD) was applied for detection and analysis.

### 2.2 Co‐culture of dual‐directional induction of hUCMSCs

For the co‐culture of the dual‐directional induction of hUCMSCs, a series of culture media were selected (Table 1). When cell confluence reached 70%‐80%, the culture medium would be changed from the proliferation medium (PM) to the induction medium. Before sent for co‐culture, the os‐hUCMSCs (osteogenically induced hUCMSCs) and en‐hUCMSCs (angiogenically induced hUCMSCs) were pre‐induced for 3 days. A gradient ratio was selected for co‐culture study with different culture medium (Table 2). For co‐culture, pre‐induced os‐hUCMSCs and en‐hUCMSCs were mixed with different ratios accordingly and incubated for another 3 days before in vitro and in vivo study.

**Table 2:** Cell ratios and culture medium used for co‐culture experiments

No.	Cell types	Cell ratios	Culture medium			
			1	2	3	4
1	os‐hUCMSCs: en‐hUCMSCs	4:0			OM	
2	os‐hUCMSCs: en‐hUCMSCs	3:1	PM	OM	EMM (2:2)	PMM (3:1)
3	os‐hUCMSCs: en‐hUCMSCs	2:2	PM	OM	EMM (2:2)/ PMM (2:2)	
4	os‐hUCMSCs: en‐hUCMSCs	1:3	PM	OM	EMM (2:2)	PMM (1:3)
5	os‐hUCMSCs: en‐hUCMSCs	0:4			EIM	

### 2.3 Determination of osteogenesis and angiogenesis capacity of co‐cultured hUCMSCs

Proliferation study (n = 6): For the observation period of 1 to 7 days, the cultured cells were incubated with the culture medium and Alamar Blue (Invitrogen) (v/v = 10:1) for 3 hours before examined at 570 nm and 600 nm for the optical density (OD). The percentage of proliferation was calculated by the following formula:

(%)=[117216×A570-80586×A600(sample)]/[117216×A570-80586×A600(control)]×100%

ALP activity (n = 4): ALP quantitative and qualitative study was both carried out. According to the manufacturer's instructions of ALP quantitative kit (Nanjing Jiancheng), after cell lysis and centrifugation, 30 μL supernatant of each sample reacted with 50 μL buffer solution and 50 μL matrix solution at 37°C for 15 minutes. Subsequently, 150 μL colour developer was added and immediately sent for the OD reader at 520 nm. The protein concentration of each sample was determined by the BCA protein quantitative kit (Best Bio) for normalization. The ALP activity was expressed as King unit per gram protein. For ALP staining, the diethyl 2,5‐di(thiophen‐2‐yl)terephthalate, 5‐bromo‐4‐chloro‐3‐indolyl phosphate (BCIP) and nitro blue tetrazolium (NBT) were mixed with the ratio of 300:1:2 to get BCIP/NBT staining working reagent (Beyotime). All the samples were fixed in 4% paraformaldehyde (PFA) for 15 minutes before incubated with the staining working reagent for 20 minutes.

Alizarin red staining (ARS) (n = 4): After sample fixation, the alizarin red solution (0.2%, pH 8.3) was added in for reaction of 10 minutes. Before imaged under the stereomicroscope to visualize the mineralization deposition, the wells were rinsed with distilled water to removing the non‐specific staining. For quantification analysis, 10% hexadecyl pyridinium chloride monohydrate (CPC) was used to dissolve the mineralized nodules and then measured the colorimetric absorbance at 562 nm.

Matrigel angiogenesis assay (n = 5): To investigate the angiogenesis capacity, the matrigel angiogenesis assay was performed. Briefly, 10 μL ice‐cold matrigel (354230, BD Biosciences) was added into the angiogenesis slides (ibidi) and sent for incubation at 37°C for 30 minutes. After that, 50 μL cell suspension (2 × 10^4^ cells) was dropped into each well and imaged after 2.5 hours incubation for microtubule formation.

Acetylated low‐density lipoprotein labelled with Dil (Dil‐ac‐LDL) phagocytosis test (n = 5): Before testing, the culture medium was changed to EIM containing 10 μg/mL Dil‐ac‐LDL (Invitrogen). After incubation at 37°C for 4 hours followed by several PBS washes, the nuclei staining was carried out with 5 μg/mL Hoechst (Sigma‐Aldrich). The confocal laser scanning microscope (CLSM, Leica) was used for observation.

Immunofluorescent staining (n = 3): After endothelial induction, the cells were fixed before penetrated by the frozen methanol at 20°C for 10 minutes. Blocking buffer (Beyotime) was employed to block the endogenous peroxidase at room temperature for 1 hour. Monoclonal antibody for EphrinB2 (1:100; Abcam) and EphB4 (1:800; CST) against human were incubated at 4°C overnight followed by incubation with secondary antibody (1:750; Biotium) for 1 hour. Later on, 5 μg/mL Hoechst (Sigma‐Aldrich) was used for nuclei staining for 15 minutess and photographed by CLSM.

Quantitative reverse transcription‐polymerase chain reaction (qRT‐PCR) (n = 3): Total RNA was extracted from the target cells by TRIzol (Invitrogen) and quantified by NanoDrop 2000 Spectrophotometer (Thermo Fisher Scientific, USA). About 500 ng of total RNA from each sample was used to synthesize complementary DNA (cDNA) via PrimeScript™ RT Master Mix (Takara). Finally, 10 ng cDNA from each sample was utilized for qPCR with TB Green Premix Ex Taq II (Takara) and synthesized primers (Generay) in real‐time fluorescent quantitative PCR (Bio‐Rad). The relative gene expression was calculated using 2^−∆∆CT^ and GAPDH as the housekeeping gene. The primer sequences were listed in Table 3. The genes examined were ALP, runt‐related transcription factor 2 (RUNX2), collagen type 1 (COL1), osteopontin (OPN), EFNB2, EPHB4, VEGF, basic fibroblast growth factor (bFGF), SERPINF1, ANGPTL1, SPROUTY1, angiogenin (ANG), CD146, PDGFB and GAPDH.

**Table 3:** Primers sequences used for qRT‐PCR analysis

No.	Transcript	Sequences
1	ALP	sense: GGCTGTAAGGACATCGCCTA antisense: GGGTCAAGGGTCAGGAGTTC
2	RUNX2	sense: TACTATGGCACTTCGTCAGGA antisense: GATTCATCCATTCTGCCACTA
3	COL1	sense: CGATGGATTCCAGTTCGATGTGGT antisense: TGTTCTTGCAGTGGTAGGTGATG
4	OPN	sense: GCTAAACCCTGACCCATC antisense: CTTTCGTTGGACTTACTTGG
5	EFNB2	sense: CTCCTCAACTGTGCCAAACCA antisense: GGTTATCCAGGCCCTCCAAA
6	EPHB4	sense: GATGCCTGGAGTTACGGGATTG antisense: TCCAGCATGAGCTGGTGGAG
7	VEGF	sense: GGAGGCAGAGAAAAGAGAAAGTGT antisense: TAAGAGAGCAAGAGAGAGCAAAAGA
8	bFGF	sense: AGTCTTCGCCAGGTCATTGAGATC antisense: CGTCCTGAGTATTCGGCAACAG
9	SERPINF1	sense: ATAGTCCAGCGGGAAGGT antisense: TAGCAAGATCACAGGCAAAC
10	ANGPTL1	sense: TACCTCCCAGCAGACCAG antisense: TTTTCCCGACAAGACACC
11	SPROUTY1	sense: ATTGCCCTTTCAGACCTA antisense: AATAATAACTACGAGCACAGAC
12	ANG	sense: TGGTGACCTGGAAAGAAG antisense: GCACTATGATGCCAAACC
13	CD146	sense: GAGACAGGTGTTGAATGCACG antisense: TGTTGGCTCTGGTATGAGGAC
14	PDGFB	sense: ATGGAGTTTGCTGTTGAGGTGG antisense: GCAGGGTGGAGGTAGAGAGATG
15	GAPDH	sense: GCACCGTCAAGGCTGAGAAC antisense: TGGTGAAGACGCCAGTGGA

Western blot (n = 3): For cell lysis, RIPA containing 1% proteinase inhibitor, cocktail (ComWin Biotech) was applied followed by centrifugation. The supernatant was harvested and quantified by the BCA kit before subjected to Western blot analysis. The protein (20 μg) was separated by 10% SDS‐PAGE gel electrophoresis, and the targeted protein was transferred to PVDF membrane and blocked with 1%. PBST containing 3% FBS for 1 hour. The primary antibody rabbit anti‐human RUNX2 (1:1000; CST), mouse anti‐human CD146 (1:1000; CST), rabbit anti‐human β‐actin (1:1000; CST) were incubated at 4°C overnight followed by the incubation of the secondary antibody goat anti‐rabbit (1:2000; CST) or rabbit anti‐mouse (1:2000; CST) for 1 hour. Finally, chemiluminescence detection reagents (ECL) was dropped onto the membrane and exposed to X‐ray film. The gel imaging system (Bio‐Rad) was used for photography, and the final results were analysed by Image J.

### 2.4 Construct and characterizations of tissue‐engineered bone graft

Three‐dimensional printing TCP scaffold fabrication: Briefly, β‐TCP (Kunshan Chinese Technology New Materials Co., Ltd.) ink was prepared by dispersing the powder in dispersant with distilled water, a proper amount of hydroxypropyl methylcellulose, and polyethylenimine (PEI) to increase agglomeration. A bio 3D printer (Regenovo) was employed for the scaffold's fabrication. The printing parameters were set as 700 μm in distance and the height of 200 μm to get the final scaffold with a diameter of 5 mm and a thickness of 1 mm. After printing, the scaffolds were dried in air for 24 hours and sintered at 1100°C for 3 hours.

For the construct of tissue‐engineered bone graft, before cells seeding, the sterile 3D TCP scaffolds were soaked in culture medium overnight and dried in air. About 15 μL cell suspension (6.67 × 10^6^/mL) of the 3:1 group was dropped onto the scaffolds. After 3 hours attachment, the culture medium was refilled.

Scanning electron microscopy (SEM): The morphology of the scaffolds and the attached cells was observed by SEM (S3400N, Hitachi). Before analysis, 2.5% glutaraldehyde (Leagene) was applied for fixation. With gradient alcohol dehydration, dry in air and gold spraying, the samples were mounted in for SEM.

Live/dead staining (n = 3): 3 days after cell seeding, the live/dead staining kit (BestBio) was used for characterization according to the instructions, and the samples were observed under CLSM.

### 2.5 In vivo study and analysis

With the permission of Guangzhou Medical University Ethics Committee, 12 male Sprague‐Dawley rats (12 weeks, weight of 250‐300 g) were chosen for the bone repair study. Groups division: blank, scaffold, 4:0 and 3:1 group (4:0 and 3:1 group were chosen based on the above research results) (n = 6). Sodium pentobarbital (30 mg/kg) was used for general anaesthesia by intraperitoneal injection. After shaved and sterilized, the soft tissue was cut along the middle line of the rat skull. The muscles and periosteum were separated bluntly to expose the operation area thoroughly. The full‐thickness bone defect with a diameter of 5 mm was made with trephine under saline irrigation continuously. Two critical‐sized calvarial bone defects were created per rat. The bone grafts were implanted in accordingly with strict sutures. Penicillin 400 000 U was injected intramuscularly for 3 days after the operation. Four weeks later, the rats received euthanasia with inhalation anaesthesia of isoflurane (Yipin China), and the specimens were harvested and fixed for further studies.

Micro CT: Micro CT (SkyScan 1172; Bruker) was used for radiography analysis with the scanning parameter of 1 mm thick aluminium filter, resolution 2000 × 1332, pixel size 9 μm, voltage 80 kV, current 100 μA. NRecon 1.0 software was then used to rebuild, Dataviewer for ROI selection and CTAn1.13 software for new bone volume, bone trabeculae number and thickness analysis. On account of no bone formed in the centre area of the defect in the blank group, the new bone volume, bone trabeculae number and thickness of the blank group were set zero.

Histology: For detecting the tissue structure of the umbilical cord, the harvested tissue was fixed in 4% PFA for 24 hours. Together with the decalcified calvarial specimens, the harvested umbilical cord tissue was washed overnight, dehydrated gradient, embedded in paraffin and sliced at a thickness of 4 μm for further studies.

For immunohistochemical staining, the umbilical tissue was sent for CD31 and CD34 staining. After pre‐treatments of the samples, mouse anti‐human CD31 (1:1600; CST) and rabbit anti‐human CD34 (1:200; Abcam) were incubated at 4°C overnight, respectively. After rewarming and PBS washing, the secondary antibody rabbit anti‐mouse (1:200; CST) and goat anti‐rabbit (1:200; CST) were added for a reaction at 37°C for 1 hour. About 10% DAB was used for colouring with haematoxylin to stain the nuclei.

For haematoxylin‐eosin (HE) staining, an automatic dyeing machine (Sakura) was used. The staining kit of Masson trichrome was applied for further analysis by the manufacturer's instructions (Servicebio). Briefly, after dewaxing, a series of staining reagents were used with haematoxylin dyeing for 3 minutes, acid fuchsin for 5‐10 minutes, molybdenum phosphate acid dipping for 1‐3 minutes and aniline blue dyeing for 3‐6 minutes. For quantitative analysis, the percentage of the new bone volume was calculated with the new bone area/ tissue area × 100%.

### 2.6 Statistical analysis

All the presented data were expressed as the mean ± standard deviation. Tukey's multiple comparisons test in one‐way ANOVA was used to a comparison of two groups, and Sidak's multiple comparisons test was used to analyse the mean of multiple comparisons tests at multiple time points. The significance level was set at *P* < .05.

## 3 RESULTS

### 3.1 Characteristics of the umbilical cord and hUCMSCs from Wharton's jelly

**Figure 1:**
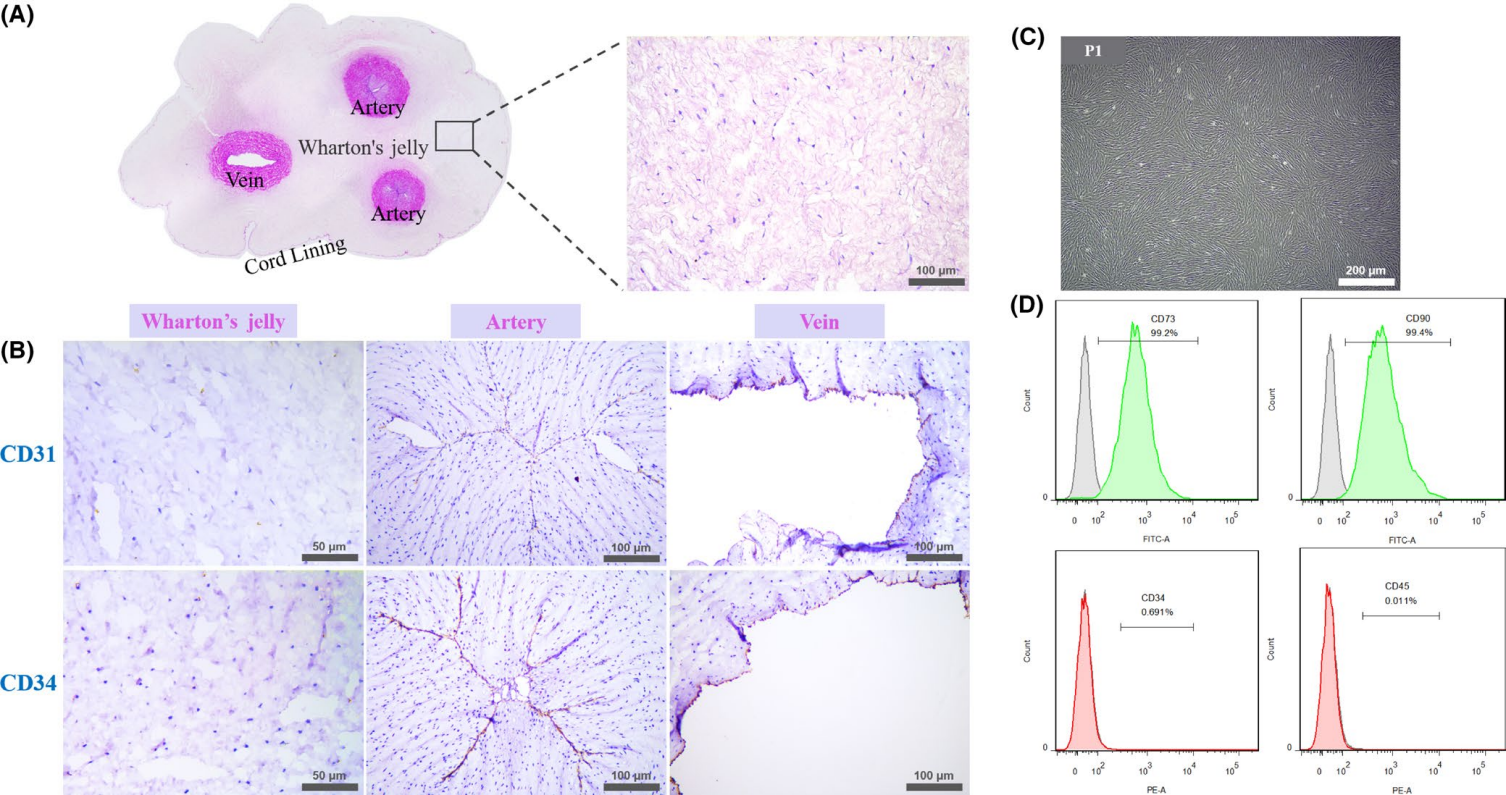
Characteristics of the umbilical cord and hUCMSCs from Wharton's jelly. A, HE staining of the umbilical cord tissue. Light pink stood for the Wharton's jelly and dark pink for the arteries and the vein. Higher magnification revealed loose and irregular jelly‐like tissue with nuclei stained dark blue; B, Immunohistochemical staining of the Wharton's jelly, arteries and vein with CD31 and CD34. Positive brown stained cells could only be detected in the arteries and vein; C, Wharton's jelly‐derived P1 cells with homogeneous bipolar spindle‐like shape, lining as a whirlpool; D, Surface markers of P5 cells

The umbilical cord tissue structure was demonstrated by HE staining. Two darkly stained round arteries coupled with one oval‐shaped vein were observed clearly in the central part of the pink‐stained Wharton's jelly, with a thinner layer epithelium surrounded. Under higher magnification, Wharton's jelly was loose with eosinophilic stained mononuclear cells scattered (Figure 1A). Immunohistochemical staining showed that Wharton's jelly did not contain CD31 and CD34 positive haematopoietic cells which could be observed clearly in the arteries and the vein with aligned brown colour (Figure 1B). The isolated P1 cells from the Wharton's jelly were homogeneous bipolar spindle‐shaped and arranged in a whirlpool (Figure 1C). Flow cytometry showed that more than 99% of the cells expressed the surface markers CD73 and CD90 of MSCs, but did not express the haematopoietic markers CD34 and CD45 (Figure 1D).

### 3.2 Osteogenic differentiation of hUCMSCs

**Figure 2:**
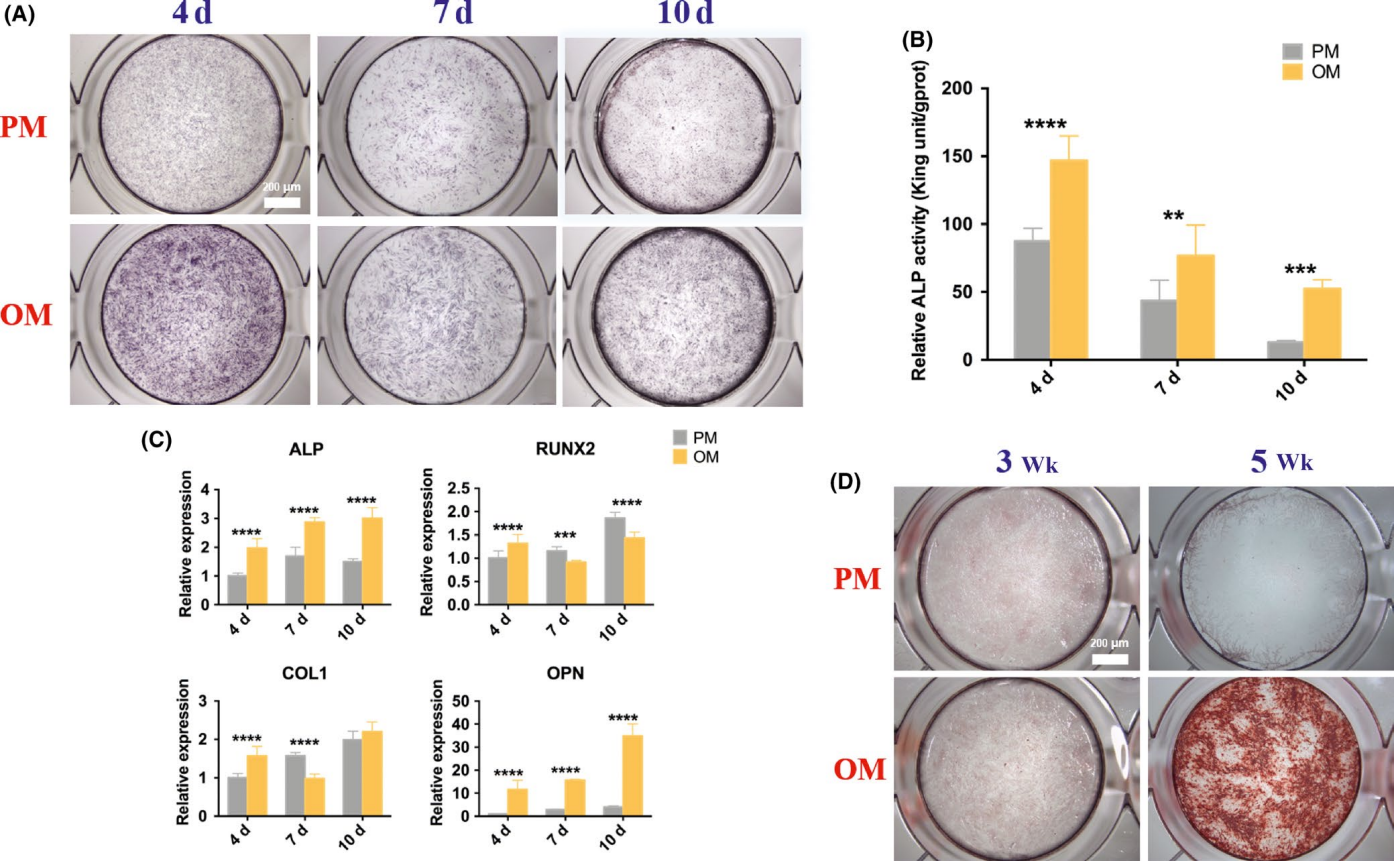
Osteogenic differentiation of hUCMSCs. A, ALP staining of non‐ and osteogenically induced hUCMSCs for 4, 7 and 10 d and (B) ALP quantitative analysis; C, The expressions of osteogenesis‐related genes ALP, RUNX2, COL1 and OPN after 4, 7 and 10 d induction; D, ARS of non‐ and osteogenically induced hUCMSCs for 3 and 5 wk. Much more red‐stained depositions observed in OM in the 5th wk. *P < .05; **P < .01; ***P < .001; ****P < .0001

To evaluate the osteogenic differentiation capacity of hUCMSCs, ALP activity of quantitative and qualitative analysis, osteogenesis‐related genes expressions and mineralized nodules were studied. After 4, 7 and 10 days of osteogenic induction, ALP staining of hUCMSCs in OM became purple, darker than that in PM, with the darkest colour observed on the 4th day (Figure 2A), which was in accordance with quantitative examination of ALP activity (Figure 2B), showing that the ALP activity increased significantly during the observed period in OM compared with that in PM, and reached the highest level on the 4th day, which was twice and three times higher than that of the 7th and 10th day, respectively. Meanwhile, the mRNA expressions of ALP and OPN increased significantly after osteogenic induction at all detection time points and gradually increased with time, while the expressions of RUNX2 and COL1 genes increased significantly only on the 4th day (Figure 2C). ARS showed scattered calcium nodules at the 3rd week after induction and patches after 5 weeks. No calcium nodules were found in the PM group (Figure 2D).

### 3.3 Endothelial differentiation of hUCMSCs

**Figure 3:**
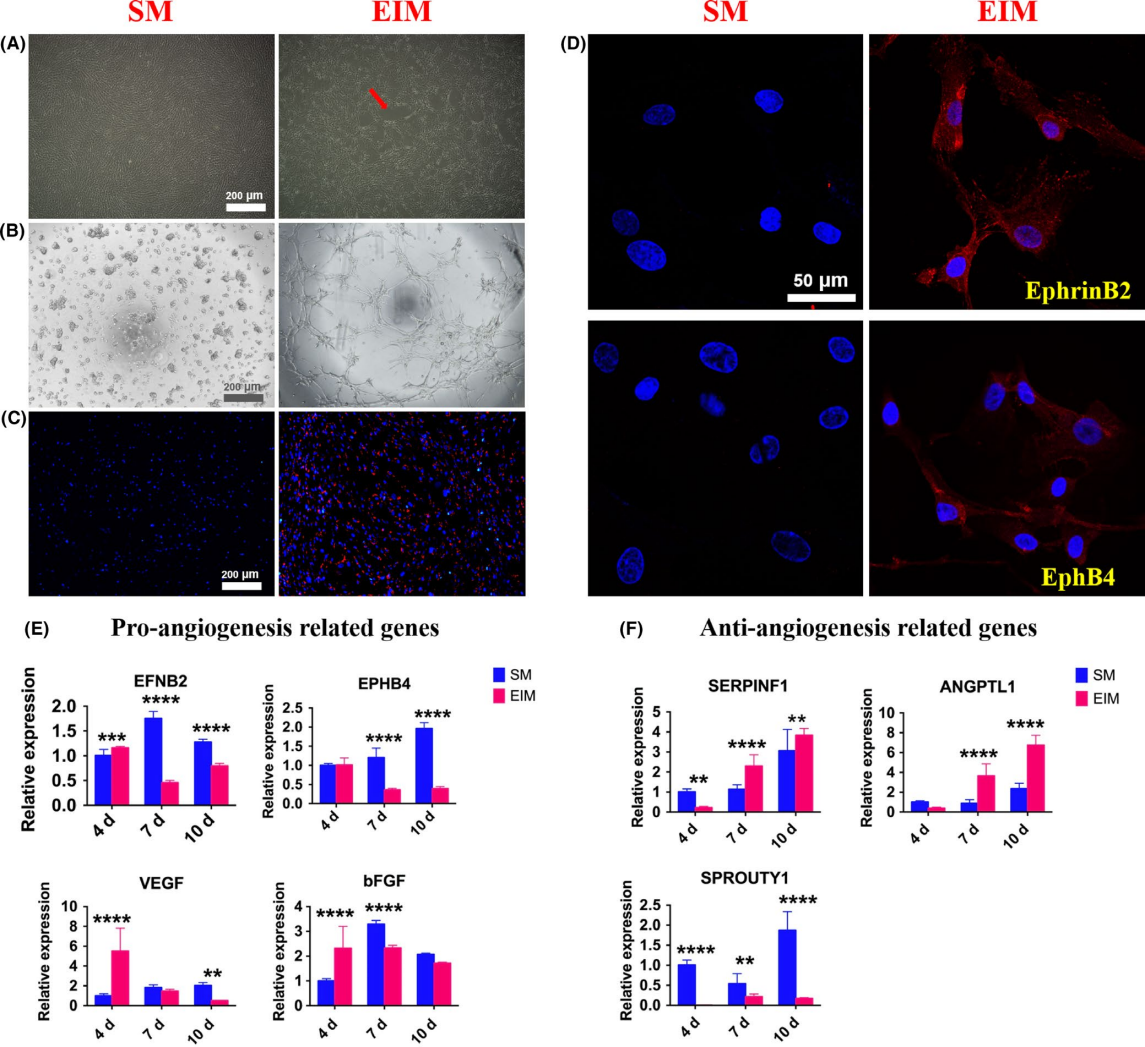
Endothelial differentiation of hUCMSCs. The morphology of induced hUCMSCs was shown as (A) vascular‐like structure indicated by a red arrow under phase contrast microscopy, with (B) angiogenesis in the matrigel and (C) Dil‐ac‐LDL phagocytosis, with blue stained nuclei and red‐stained Dil labelled ac‐LDL; D, Immunofluorescence assay validated the expressions of EphrinB2 and EphB4 proteins; E, The expressions of pro‐angiogenesis‐related genes EFNB2, EPHB4, VEGF and bFGF, coupled with (F) anti‐angiogenesis‐related genes SERPINF1, ANGPTL1 and SPROUTY1 after 4, 7 and 10 d induction. *P < .05; **P < .01; ***P < .001; ****P < .0001

Three days after EIM induction, the induced hUCMSCs became elongated and polygonal with a large number of vascular‐like structures (Figure 3A). Since tube formation on the matrigel and ac‐LDL phagocytosis is the two representational experiments to determine whether the induced MSCs possess endothelial cells’ functions, we examined the morphology and function changes with the two tests. Studies showed that the 4 days’ pre‐induced hUCMSCs inoculated on the matrigel for 2.5 hours could form a reticular structure like vessels, while normal hUCMSCs distributed in clusters (Figure 3B). Similarly, pre‐induced hUCMSCs could engulf Dil‐ac‐LDL, with red fluorescent‐labelled ac‐LDL found around the blue nuclei, while the non‐induced MSCs did not possess the same function (Figure 3C). Immunofluorescence staining showed that the cell connected in a circular pattern after induction, expressing both arterial endothelial cells’ marker EphrinB2 and vein endothelial cells’ marker EphB4 in the cytoplasm (Figure 3D). QRT‐PCR examination detected that the expressions of angiogenic genes EFNB2, EPHB4, VEGF and bFGF on the 4th, 7th and 10th day after induction, and EFNB2, VEGF and bFGF enhanced on the 4th day. All the tested genes expressions declined on the 7th and 10th day significantly (Figure 3E). In contrast, except for the expression of anti‐angiogenic gene SPROUTY1 declined significantly, the expressions of SERPINF1 and ANGPTL1 increasing over the whole period were contrary to that of EFNB2, VEGF and bFGF (Figure 3F).

### 3.4 Screening of the co‐culture medium

**Figure 4:**
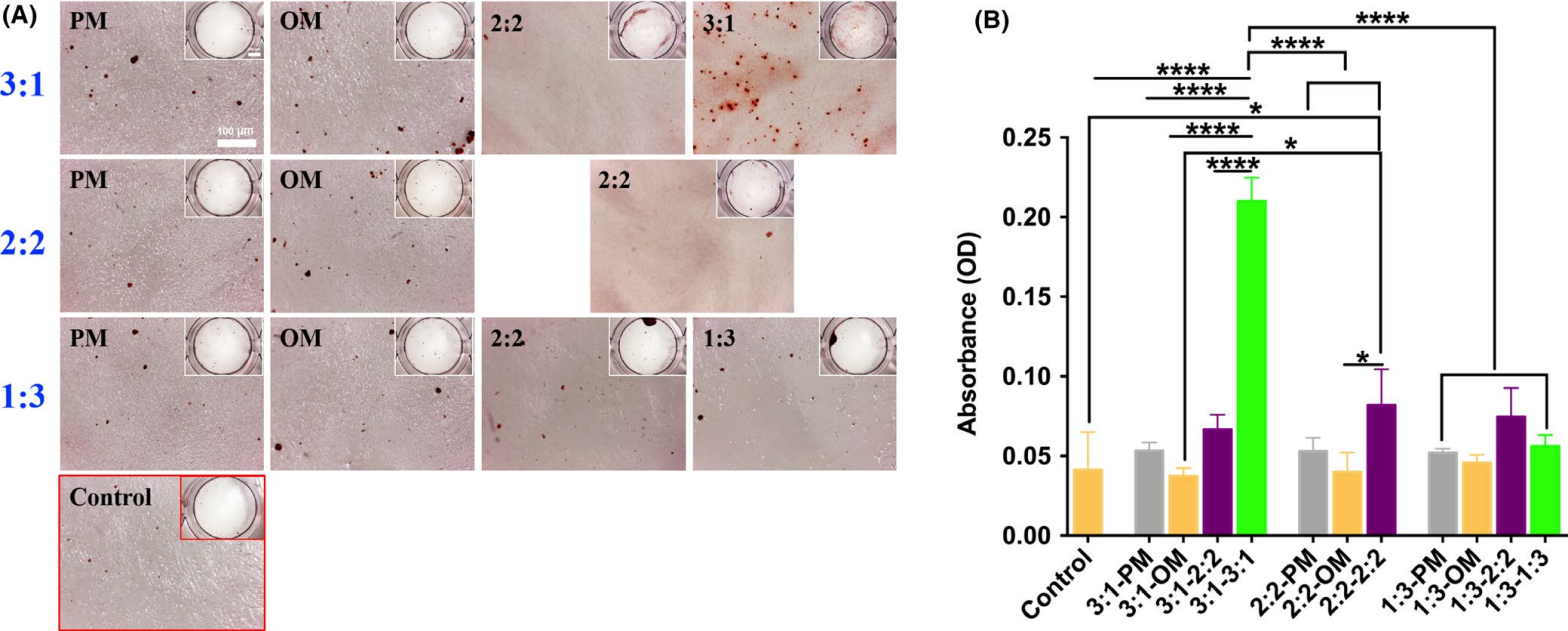
Screening the co‐culture medium for bi‐directional induction of hUCMSCs. A, ARS results of pre‐induced os‐hUCMSCs and en‐hUCMSCs co‐cultured under gradient ratios and different medium for 17 d, and osteogenically induced hUCMSCs in OM was set as the positive control; B, Calcium nodules quantification assay. *P < .05; **P < .01; ***P < .001; ****P < .0001

When pre‐induced os‐hUCMSCs and en‐hUCMSCs were co‐cultured for 17 days, ARS showed that cells co‐cultured in PM with the ratio of 3:1, 2:2 and 1:3 also had osteogenic differentiation ability with calcium nodules formation (Figure 4A), which had no statistical difference with the positive control group (Figure 4B). Similarly, there was no significant difference between PM, OM, EMM (2:2) groups and control group. Only in PMM (3:1) culture medium, a large number of scattered calcium nodules formed in 3:1 group (Figure 4A), which was dramatically increased compared with the others with the OD value of about 5 times higher than that of the control group (Figure 4B). Therefore, PMM (3:1) was selected for the subsequent co‐culture medium.

### 3.5 Effects of co‐culture on the osteogenesis and angiogenesis of hUCMSCs

**Figure 5:**
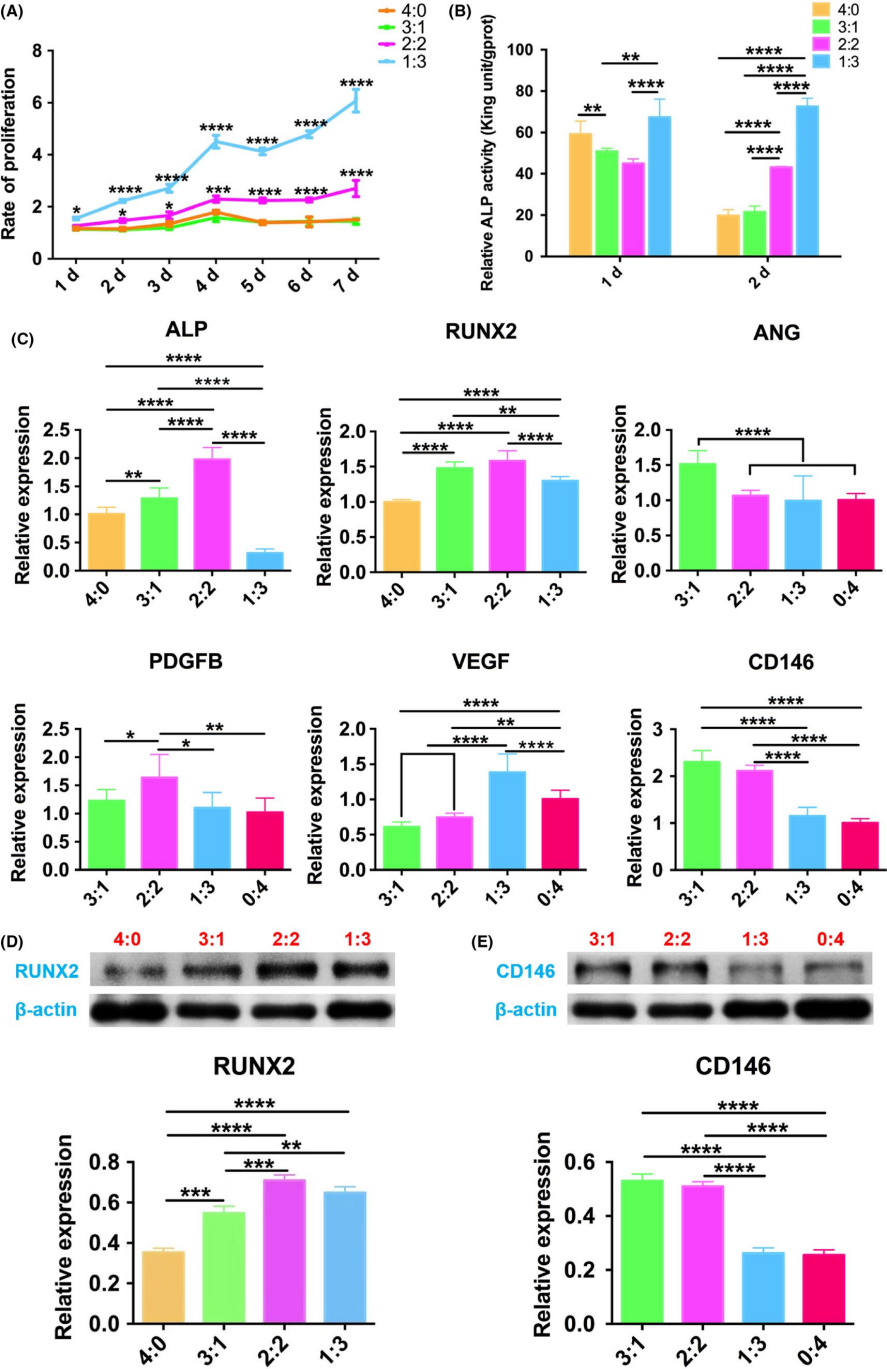
Co‐culture of hUCMSCs of bi‐directional pre‐induction. A, Cell proliferation curve; B, ALP quantitative study; C, Expressions of osteogenesis‐ and angiogenesis‐related genes after co‐culture for 3 d; D, RUNX2 protein expression after co‐culture for 3 d and (E) CD146 protein expression. *P < .05; **P < .01; ***P < .001; ****P < .0001

After co‐culture, the cell proliferation rate and ALP activity of 3:1 group were similar to those of 4:0 in OM, which was the positive control (Figure 5A,B), while the osteogenesis‐related genes RUNX2, ALP and protein expression in group 3:1 were higher than that of 4:0 (Figure 5C,D). The proliferation rate of 2:2 and 1:3 group maintained at a higher standard in the overall observation (Figure 5A). Considering the osteogenesis‐related genes and protein expression, ALP, RUNX2 and its protein of 2:2 group were the highest (Figure 5C,D). In contrast, ALP activity of 1:3 group on the 1st and 2nd day showed the highest compared with the others (Figure 5B), while ALP gene expression decreased after 3 days’ co‐culture, with RUNX2 gene and its protein still higher than that of 4:0 group (Figure 5C,D).

For angiogenesis analysis, compared with 0:4 group, ANG mRNA expression of 3:1 group was the highest on the 3rd day, as well as PDGFB mRNA expression of 2:2 group, VEGF mRNA expression of 1:3 (Figure 5C). All these differences were statistically significant. CD146, the marker of pericyte, related to vascular stability, its gene and protein expression declined from 3:1 group to 0:4 group gradually (Figure 5C,E).

**Figure 6:**
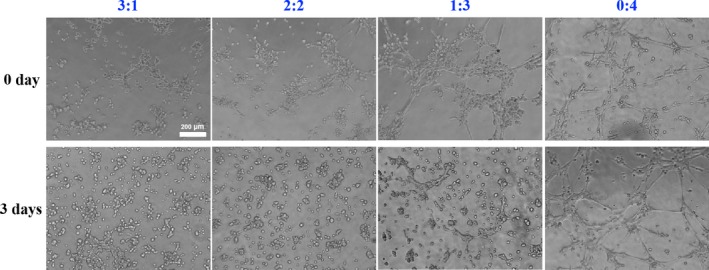
Tube formation capacity of the co‐cultured system of 3 d. The more percentage of en‐hUCMSCs mixed, the more vascular tubules formed on the matrigel, while no obvious branches or reticular structure observed in 3:1 and 2:2 groups

To further illustrate the angiogenetic capacity of the co‐culture system, the matrigel tube formation assay was adopted. After 3 days pre‐induction (0 day), the more percentage of en‐hUCMSCs mixed, the more vascular tubules formed on the matrigel, with a more complete and continuous circular shape (Figure 6). However, after 3 days co‐culture, the 0:4 group still possessed good angiogenesis ability, but the tube formation in the other groups predominantly weakened, with only a small amount of branching structures (Figure 6).

### 3.6 Characterization of the tissue‐engineered construct with co‐cultured dual‐directional differentiated hUCMSCs

**Figure 7:**
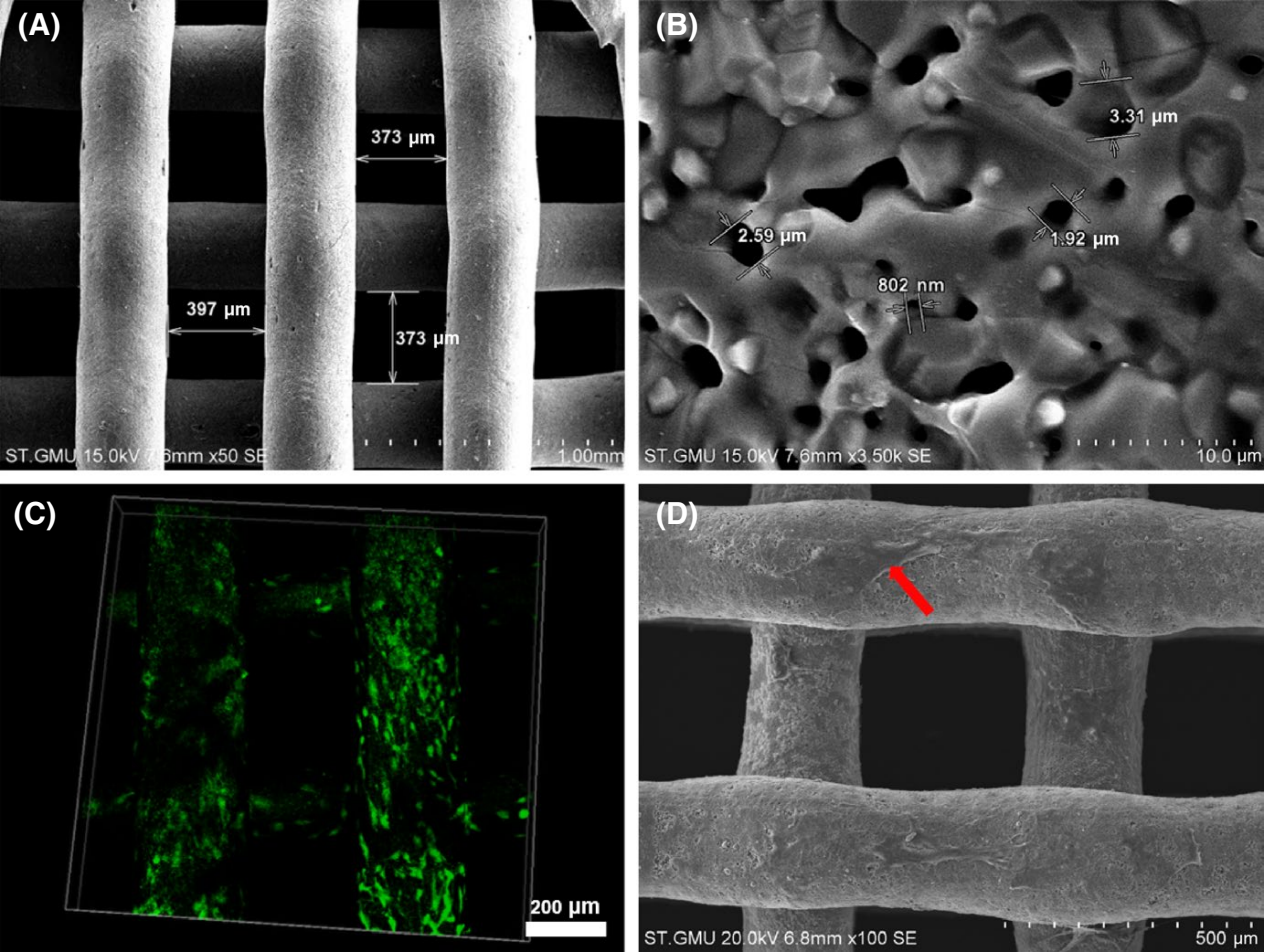
Characterization of the tissue‐engineered construct with co‐cultured dual‐directional differentiated hUCMSCs. A, Surface architecture and (B) microstructure of scaffold under SEM; C, Live/dead staining results showed green stained living cells attached to the scaffold; D, Dual‐directional differentiated hUCMSCs spread well onto the scaffold by SEM. Red arrow referred to dual‐directional differentiated hUCMSCs

SEM showed that the struts of the 3D printed TCP scaffolds were dedicated and uniform with pore size ranging from 350 to 400 μm (Figure 7A). At 3.5 k magnification, microporous and nanoporous structures could be observed (Figure 7B). Live/dead staining results showed that green‐stained viable cells attached to the scaffold with hardly visible red‐stained dead cells (Figure 7C). Similarly, the hierarchical porous structure was beneficial to the growth and migration of MSCs, suggesting the 3D scaffolds possessed excellent cytocompatibility, and this combination was feasible for bone graft fabrication (Figure 7D).

### 3.7 Rehabilitation of the critical‐sized bone defect

**Figure 8:**
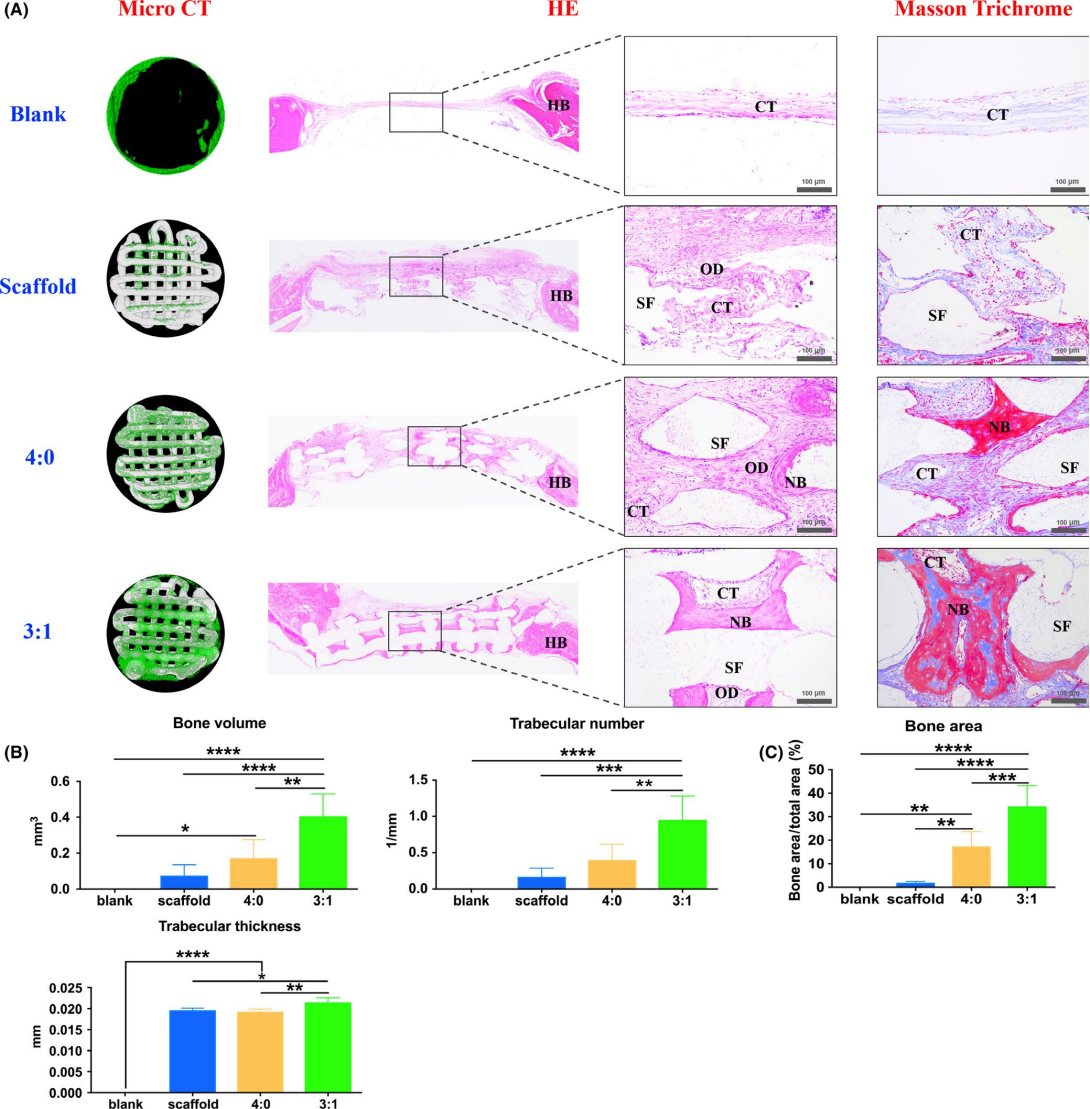
In vivo rehabilitation outcome of rat's calvarial bone defect. A, Micro CT and histology outcome validating the reconstruction outcome of different approaches. For micro CT, green‐stained tissue referred to the newly formed bone, while the grey stood for the 3D scaffold. For histology analysis, pink‐stained homogeneous osteoid and new bone structure from HE staining and dark red‐stained mature bone and light blue‐stained collagen tissue from Masson trichrome staining could be detected in the 3:1 group. CT: connective tissue; SF: scaffold; OD: osteoid; NB: new bone; HB: host bone; B, Quantitative comparison of new bone volume, trabecular number and thickness, respectively; C, Percentage of new bone by histological study. *P < .05; **P < .01; ***P < .001; ****P < .0001

After the operation, all rats recovered soundly. From micro CT, 4 weeks post‐operation, only a small amount of new bone grew from the host bone in the peripheral area of the defect in the blank group. With scaffold implantation, less bone formed on the scaffold. When incorporating the os‐hUCMSCs, much new bone grew evenly along the scaffold. Moreover, the 3:1 ratio of os‐hUCMSCs to en‐hUCMSCs could significantly enhance the bone volume, trabecular number and thickness (Figure 8A‐B).

For further ensuring the bone formation outcome, HE and Masson trichrome staining were both applied. From the gross view of the newly regenerated tissue in the defect area, amounts of pink‐stained connective tissue filled in with slightly new bone regenerated along the host bone. By scaffold implantation, there were observed osteoid in the central part with a few capillaries. With os‐hUCMSCs coated scaffold implantation, red‐stained mature bone regenerated in the central area with dense, regular connective tissues along the porous structures of the decalcified TCP scaffold. In group 3:1, abundant mature bone scattered with dark pink‐stained bone lacuna surrounded by less connective tissue (Figure 8A). With Masson trichrome staining, dark red‐stained mature bone and light blue‐stained collagen could be observed in the 3:1 group, while less mature bone formed in 4:0 group and no bone regenerated only with or without scaffold. The quantitative study of histology also validated that the 3:1 group possessed a greater bone inductive capacity (Figure 8C).

## 4 DISCUSSION

With increasing demands of bioactive bone grafts in clinical settings, MSCs have been regarded as an ideal approach compared with cytokines in addition which often bring about unexpected side effects for patients, to enhance the osteogenic, angiogenic, immunoregulatory capacity of bio‐scaffolds. Considering that larger the bone scaffolds, harder the seeded cells to live inside for the limited perfusion and migration of capillaries, in this study, dual‐directional differentiations of hUCMSCs were conducted, coupled with enhanced osteogenic capacity with co‐culture strategy, so as to obtain osteoblasts and endothelial cells’ functions in fabricating bioactive tissue‐engineered bone graft for critical‐sized bone defects rehabilitation.

In terms of osteogenic differentiation, the results of the two‐dimensional induction culture showed that hUCMSCs were much weaker than that of hBMSCs.^12^ After culture in OM, the ALP activity and osteogenic genes (ALP, RUNX2, COL1 and OPN) expressions level of hUCMSCs were higher than those without induction, while ARS showed that only dispersedly punctiform calcium nodules appeared on the 3rd week, consistent with studies reported before,^25^, ^26^, ^27^ and flaky calcification did not occur until the 5th week, suggesting that hUCMSCs could achieve a similar outcome of osteogenesis to hBMSCs with a longer induction time. This phenomenon might result from the different origins of the two kinds of cells. That is, hBMSCs are more likely to express the same genes as osteoblasts, while hUCMSCs tend to express the same genes as embryonic stem cells.^25^ Batsali et al found that most of the genes positively related to WNT signalling pathway were significantly reduced in hUCMSCs during osteogenic differentiation compared with hBMSCs for WNT‐1 inducible‐signalling pathway protein‐1 (WISP1) could be used to induce the increased expressions of osteogenesis‐related genes significantly.^27^ Thus, it is speculated that the inadequate activation of WNT signalling pathway in hUCMSCs may lead to relatively lower osteogenesis ability.

For angiogenic induction, reported methods included cytokine‐induction,^28^ hypoxia‐induction,^29^, ^30^ co‐culture induction^31^, ^32^ and gene transfection‐induction.^33^ Angiogenesis‐related cytokines‐induced differentiation of MSCs into endothelial cells are the most commonly used and currently a more successful approach. Herein, EIM was prepared by adding VEGF and bFGF into EGM. After 4 days induction, tube formation and phagocytosis characterization of endothelial cells were found in pre‐induced hUCMSCs, which was in accordance with other reports.^34^, ^35^ Meanwhile, the expressions of EphrinB2, a specific marker of the artery, and EphB4 protein specific for the vein increased.^28^, ^36^ However, compared with the control group, the relative angiogenic genes expressions of EFNB2, EPHB4, VEGF and bFGF increased on the 4th day and decreased on the 7th and 10th day, while the anti‐angiogenic genes expressions of SERPINF1 and ANGPTL1 showed the opposite outcome. Only, the expression of SPROUTY1 was lower than that of the control group. Similarly, Konig et al confirmed that human amniotic stromal cells (hAMSCs) acquired some angiogenesis characteristics, but the genes and protein microarray examinations found that the angiogenic genes expressions decreased significantly, while anti‐angiogenic genes expressions increased accordingly in angiogenic induction study.^37^ It might be concluded that after angiogenic induction, hUCMSCs could prevent themselves from differentiating into mature endothelial cells by up‐regulating the expressions of anti‐angiogenic genes. Considering the reasons why our results differed from other researches focusing on inducing endothelial cells differentiation of MSCs, presenting that the expressions of angiogenic genes in MSCs increased significantly after endothelial cells induced differentiation,^35^, ^38^ the purity and endothelial differentiation ability of MSCs are directly determined by the cell types and sources. For instance, bone marrow and fat are highly vascularized tissues. It is difficult to ensure that there are no endothelial or endothelial precursor cells included during hBMSCs/hADMSCs isolation.^37^ In contrast, no capillaries exist in the Wharton's jelly, from which the hUCMSCs derived. hADMSCs secrete amounts of angiogenesis‐related proteins,^26^ which can also affect their endothelial differentiation. Furthermore, the composition of the induction medium is different though we all adopted VEGF as the main component. The concentration of VEGF may directly affect the results of the study proved by Chen et al^35^ and Xu et al^34^ that the VEGF concentration of 50 or 100 ng/mL is more likely to cause changes in angiogenesis‐related genes, proteins and functions, while lower concentration of VEGF might not be enough to achieve this effect as VEGF is crucial to trigger vascular development and the formation of blood vessels, with VEGFR2 of osteoblasts mediating VEGF's final functions in osteogenesis and vascular endothelial cell biology.^37^, ^39^ Konig et al just utilized EGM‐2 medium for induction of hADMSCs,^37^ while 25 ng/mL VEGF and 10 ng/mL bFGF were added to EGM‐2 to induce hUCMSCs in our study, which showed that there were no consistent changes in angiogenesis‐related genes, proteins and functions in a short period of time. Similarly, no significant correlation between transcriptional factor RUNX2 and VEGF as Cbfa1/RUNX2 could regulate VEGF expression, and there was a positive feedback among RUNX2, BMP2/4 and VEGF during bone formation.^40^, ^41^ Although 1:3 group cultured in PMM (1:3) possessed more concentration of VEGF than 3:1 and 2:2 group, much higher VEGF impaired the calcium deposition, while the latter two corroborated the synergistic effect of osteogenesis and angiogenesis‐related genes expressions derived from the co‐culture of the dual‐directional differentiation of hUCMSCs. Lastly, the reported duration of induction is different, including 5,^37^ 7,^42^ 9,^34^ 12,^35^ 14 days^38^, ^43^ or a much longer period of time.

Multiple studies have shown that compared with be cultured alone, osteoblasts co‐culturing with angiogenetic cells resemble the physiological condition in vivo with synergistic effects on the process of osteogenesis and angiogenesis.^20^, ^44^, ^45^ Yang et al have attempted to induce the dual‐directional differentiation of hADMSCs into osteoblasts and endothelial cells, respectively, and the levels of osteogenesis and angiogenesis‐related genes and proteins expressions increased significantly with 1:1 co‐culture in vitro compared with 2:1 and 1:2.^42^ For further study of the co‐culture, we have extended the mix ratios coupled with the related culture medium and explored the in vivo outcome for bone regeneration. ARS was selected for screening the best co‐culture strategy. The results showed that significant differences were found among the four groups, and the 3:1 ratio of osteoblasts to endothelial cells coupled with OM to EIM culture condition exhibited much more calcium nodules. For the 3:1 group, the cell proliferation rate was much slower, which demonstrated that the co‐cultured cells entered the cell differentiation stage earlier, with the genes expressions of the earlier osteogenesis marker, ALP and RUNX2, the pro‐angiogenesis‐related ANG,^46^ and the pericyte‐associated surface marker of CD146^47^, ^48^ significantly rising, suggesting that the 3:1 co‐culture system could effectively promote physiological processes related to osteogenesis and angiogenesis, ultimately accelerating osteogenic differentiation of hUCMSCs.

For in vivo study, biocompatible 3D printed β‐TCP scaffolds^49^, ^50^ were used as carriers, and the bi‐directional pre‐induced differentiated hUCMSCs of different ratios as seed cells were combined to fabricate bioactive tissue‐engineered bone graft to repair the critical‐sized cranial bone defects of rats, which further validated that 3:1 group showed the optimal rehabilitation outcome compared with 4:0 group, in accordance with other researches. Zhou et al inoculated endothelial cells derived from rabbit BMSCs and BMSCs at the ratio of 1:1 onto β‐TCP scaffolds for repairing 1.5 cm ulnar segmental defect of rabbit with a satisfying outcome.^51^ Though the co‐culture system of bi‐directional pre‐differentiated hUCMSCs could effectively promote osteogenesis, better than that of osteogenic induction alone, the optimal pre‐induction time and co‐culture conditions need to be further studied for clinical instructions. At present, many in vitro studies have shown that there are three communication modes between osteoblasts and endothelial cells. One is soluble cytokines secreted by endothelial cells, such as bone morphogenetic protein‐2 (BMP‐2), VEGF, PDGF acting on osteoblasts in the form of paracrine to promote their differentiation.^52^ Secondly, the extracellular matrix plays a pivotal role in the communication between two kinds of cells.^53^, ^54^, ^55^ Finally, multiple scholars believe that the direct contact between cells is the premise of endothelial cells to promote the functions of osteoblasts, and gap junctions are decisive.^56^ Besides, in vivo studies of long bone have found that H‐type blood vessels are important structures for coupling osteogenesis and angiogenesis,^57^ which rely on the Notch signalling pathway of vascular endothelial cells.^58^ Therefore, in our study, whether there are the same or similar communication modes in vitro and in vivo of co‐cultured bi‐directional pre‐induced hUCMSCs is worth further studies. Nevertheless, there are still many shortcomings in this study. First and foremost, bi‐directional pre‐induced hUCMSCs should be labelled for identifying the phenotypic changes after pre‐induction and co‐culture by flow cytometry, and the cell transformation after implantation. Secondly, for instructing the clinical application of hUCMSCs, human originating cells were directly used in non‐immunodeficient SD rats, and no immunosuppressive agents were injected after the operation. Although literature says that the immunogenicity of hUCMSCs is much lower, the immune response will happen in this process inevitably. Besides, the genome and proteome of the dual‐directional differentiated hUCMSCs should be conducted and analysed for clarification of induction efficiency and further knowledge of the cells’ crosstalk. All these problems will be gradually solved in the follow‐up experiments.

## 5 CONCLUSION

Our study validated that hUCMSCs possess a slightly weaker osteogenic differentiation ability and can be induced and differentiated into endothelial‐like cells with endothelial function in a short time. The osteogenesis capacity of bi‐directional differentiated hUCMSCs into osteoblasts and endothelial cells co‐cultured in the PM was dramatically enhanced. Besides, the 3:1 group showed the optimal osteogenesis capacity both in vitro and in vivo when cultured in the same proportion of mixed medium. This co‐culture strategy of bi‐directional pre‐induction of hUCMSCs provides a novel approach for the construction of bioactive tissue‐engineered bone graft for clinical transplantation with enhanced osteogenesis.

## CONFLICT OF INTEREST

The authors declare that they have no conflicts of interest to disclose.

## AUTHORS CONTRIBUTION

Miao Zhou and Zhiyong Zhang designed the experiments and revised the manuscript. Qiong Rong and Shuyi Li carried out, analysed and wrote the manuscript draft, contributing equally to this study and sharing the first authorship. Yang Zhou, Yuanming Geng, Shangbin Liu and Wanqiu Wu assisted in performing part of the in vitro and in viv*o* study. Tim Forouzanfar and Gang Wu helped to revise the manuscript.

## Data Availability

The data that support the findings of this study are available from the corresponding author upon reasonable request.
