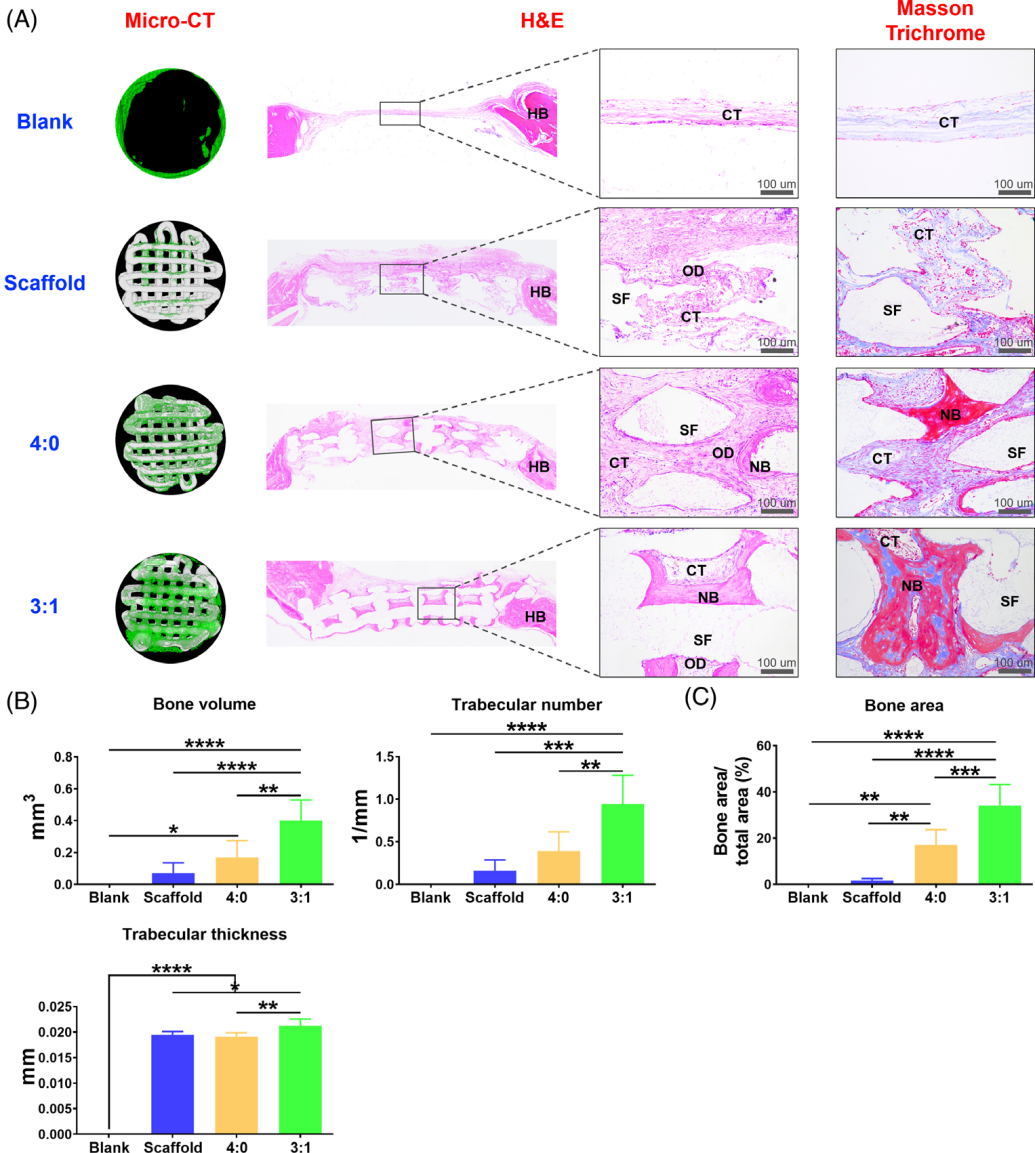# Correction

**DOI:** 10.1111/cpr.13572

**Published:** 2024-01-03

Correction to “A novel method to improve the osteogenesis capacity of hUCMSCs with dual‐directional pre‐induction under screened co‐culture conditions”

Rong Q, Li S, Zhou Y, et al. A novel method to improve the osteogenesis capacity of hUCMSCs with dual‐directional pre‐induction under screened co‐culture conditions. Cell Prolif. 2020;53:e12740. https://doi.org/10.1111/cpr.12740

The black square in Figure 8, HE staining of group 4:0 was slightly adjusted.

The corrected Figures 8 is below.

We apologize for this error.