# Supplementary material for: A novel method to improve the osteogenesis capacity of hUCMSCs with dual‐directional pre‐induction under screened co‐culture conditions
Source: Cell Prolif. 2019 Dec 9;53(2):e12740. doi: 10.1111/cpr.12740 (PMC7078770; doi:10.1111/cpr.12740)
Supplement: Supplementary file 1 [file CPR-53-e12740-s001.docx]

**A novel method to improve the osteogenesis capacity of hUCMSCs with dual-directional pre-induction under screened co-culture conditions**

***Improved osteogenesis of pre-induced MSCs co-culture***

Qiong Rong^1, 2 #^, Shuyi Li^3 #^, Yang Zhou^1^, Yuanming Geng^4^, Shangbin Liu^1^, Wanqiu Wu^1^, Tim Forouzanfar^3^, Gang Wu^5^, Miao Zhou^1 *^, Zhiyong Zhang^6 *^


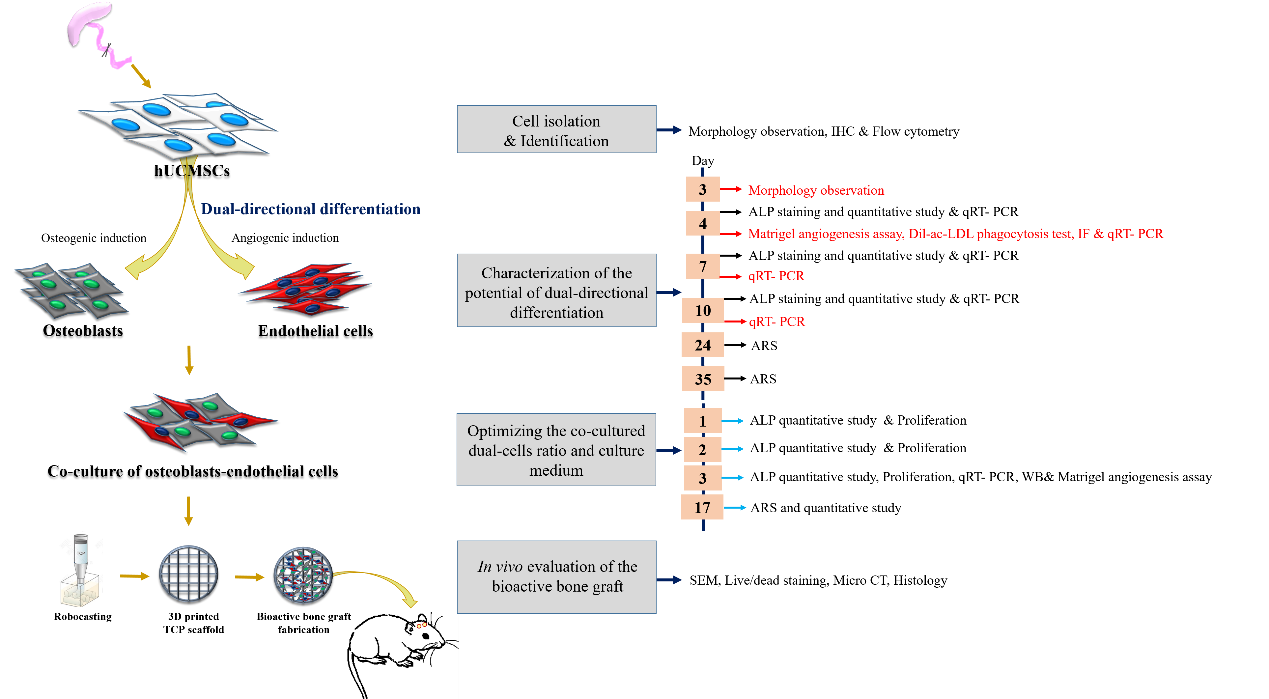


FIGURE S1 Schematic illustration of the study from hUCMSCs isolation, dual-directional induction and differentiation examination, screened co-culture condition to the final *in vivo* regeneration outcome coupled with 3D printed TCP scaffolds in rats. In the right timeline, the black, red and blue arrow represents the examinations for osteogenesis, angiogenesis, and co-culture, respectively.
